# Relationship between nurses’ knowledge of COVID-19, professional quality of life, and practice during the COVID-19 pandemic: A descriptive correlational study

**DOI:** 10.1371/journal.pone.0287457

**Published:** 2023-06-22

**Authors:** Sun Ju Kim

**Affiliations:** Chungnam National University Se-Jong Hospital, Sejong-si, Republic of Korea; University of Bern: Universitat Bern, SWITZERLAND

## Abstract

The purpose of this study was to examine knowledge, professional quality of life, and practices among nurses during the coronavirus disease 2019 (COVID-19) pandemic and explore factors associated with nurses’ practice. A total of 167 nurses were recruited from 4 general hospitals for this cross-sectional study, which was conducted from June to July 2021. Using SPSS/WIN 22, the collected data were analyzed using descriptive statistics, t-tests, analysis of variance, Pearson’s correlation coefficients, and hierarchical multiple regression analysis. The mean age of the participants was 31.43, and the sample comprised 144 women (86.2%) and 23 men (13.8%). The results indicated that practice was negatively correlated with burnout (r = -.18, p = .017). The regression model explained 24.1% of the variance. For general characteristics in Model 1, education on COVID-19 management (β = .18, p = .014) was the factor most associated with nurses’ practice. In Model 2, with professional quality of life added, burnout (β = -.21, p = .003) was the only influential factor. These results highlight the need to establish an effective prevention system for infectious diseases such as COVID-19, including education programs pertinent to the prevention and management of infection that improve the modifiable predictors of nurses’ practice—education and burnout.

## Introduction

After the reporting of the first case in Wuhan, China in December 2019 [1], the coronavirus disease 2019 (COVID-19), a communicable infectious disease, quickly escalated to a pandemic [2]. As of May 22, 2022, more than 502.2 million confirmed cases and 6 million deaths were reported, and the global excess mortality estimate for 2020–2021 was 14.9 million [3]. The pandemic has strained healthcare systems and inflicted socioeconomic damage worldwide [4].

Furthermore, as of August 5, 2021, approximately 522,219 and 1,677 healthcare professionals had contracted and died from COVID-19, respectively, in the United States [5], with approximately 5.5% of the nursing population having been infected [6]. However, these figures do not accurately reflect the actual number of healthcare personnel affected by COVID-19, as some patients only develop mild symptoms or are asymptomatic [6].

The key to preventing the spread of infection within hospitals includes the early discovery of suspected cases through close monitoring and strict adherence to isolation guidelines [7]. Studies have reported that healthcare professionals display a moderate level of knowledge and attitudes toward infectious diseases [8], suggesting that only a small fraction strictly adhere to the correct precautions [9]. In China, infection among healthcare professionals was reported to be common among frontline healthcare staff in Wuhan but not in other regions [10,11].

Negligence and lack of knowledge were pinpointed as responsible for the quick spread of infection in the early days of the pandemic [10,11], showing that a lack of knowledge among healthcare professionals can contribute to disease infection and spread. COVID-19 is highly transmissible and causes asymptomatic cases in communities [12,13]. Furthermore, an effective response to infectious diseases is hindered by a lack of knowledge about risks and response measures [14], highlighting the importance of nurses’ knowledge regarding COVID-19.

Professional quality of life (ProQOL) is defined as individual negative and positive emotions in relation to helping others who are experiencing pain or trauma. ProQOL has two components: compassion satisfaction (CS) and compassion fatigue (CF) [15]. CS is a pleasurable feeling derived from having the ability to help others [15]. CF has two components: 1) burnout associated with exhaustion, frustration, anger, and depression; and 2) secondary traumatic stress (STS), a negative emotion resulting from fear and task-related trauma [15]. Burnout refers to the development of empathy fatigue that can occur in professional caregivers while caring for various individuals [16]. STS occurs when the stress experienced in the process of maintaining close contact with the patient to help the patient gradually accumulates without being resolved [17]. Nurses’ ProQOL is an essential factor that contributes to patient care [18], and nurses’ perceived meaning of life, purpose, and values have a major impact on their own quality of life [19]. Nursing practice can be a satisfying experience based on compassion; however, compassion can also negatively affect nurses’ physical and mental health and practices [20]. Nursing professional value shows a positive correlation with pleasant feelings derived from providing care, a dimension of ProQOL known as CS, which improves clinical care quality and nurse job satisfaction [21]. Nursing professional value guides clinical practice professionally and ethically and forms nurse identity [22]. A decrease in satisfaction with compassion can lead to compassion-related avoidance behavior, which can reduce nurses’ compassion toward patients and, ultimately, their competence and quality of clinical practice. [23,24]. In Zakeri et al.’s [25] study, there was a significant correlation between clinical competency, CS, STS, and burnout, and regression analysis showed that people with high CS had strong resistance to STS and burnout and high clinical competency. In a study by Hooper et al. [26], interventions that can increase CS and lower burnout and STS are recommended to help nurses provide quality patient safety and maintain a caring attitude in practice. Burnout can cause physical and mental problems in nurses, such as insomnia, headaches, poor concentration, long-term fatigue, and irritation. This can result in poor quality of care and patient satisfaction and increased medical errors, malpractice, morbidity, and mortality [27].

According to a study comparing 2020 and 2021 data of advanced practice providers caring for patients with COVID-19, in 2021, 97.67% of respondents had been vaccinated. Despite a decrease in risk perception, stress levels remained high (2020 vs 2021 = 75.6% vs 90.7%; p = 0.09) as thoughts of resignation increased (2020 vs 2021 = 0.0% vs 11.6%; p = 0.01) [28]. Additionally, in 2021, 57% of participants reported moderate or high levels of traumatic stress and 75% reported moderate or high levels of burnout, while only 7% of participants reported high levels of compassion satisfaction [28].

Nurses are healthcare professionals who provide direct patient care and play a crucial role in the prevention and management of infections [29]. Job-related stress factors, such as CF and burnout, adversely affect their clinical decision-making [30], quality of patient care [30], clinical competency [30], and practice [31]. Although several studies have evaluated nurses’ knowledge, attitudes [32], practice [33–35], and perceptions and level of confidence [35] in the context of COVID-19, research has not comprehensively evaluated various aspects of knowledge, ProQOL, and practice.

Therefore, this study examined nurses’ knowledge, ProQOL, and practice during the COVID-19 pandemic and explored factors that influence nurses’ practice, ultimately presenting foundational data for developing measures to effectively prevent the spread of infectious diseases in the future.

## Materials and methods

### Objectives and hypotheses

**Fig 1:**
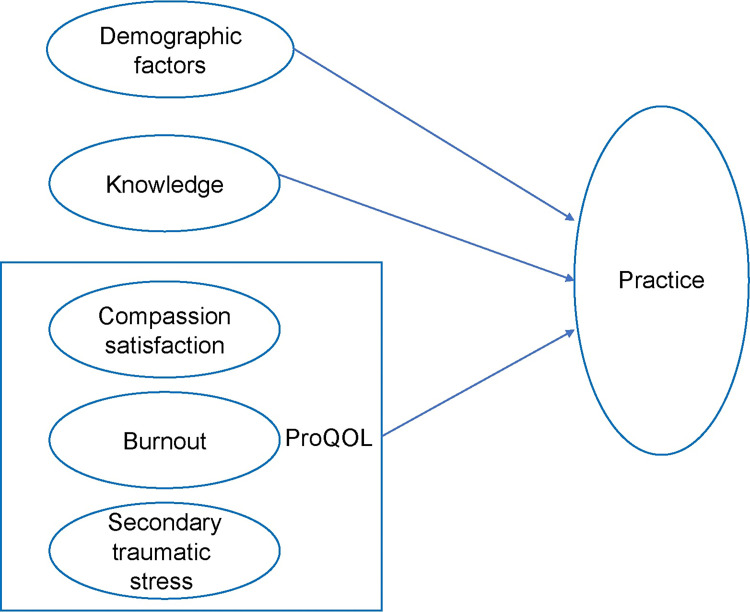
Conceptual framework for practice.

The specific objectives were as follows: 1) to examine nurses’ characteristics, knowledge, ProQOL, and practice during COVID-19; 2) to analyze the correlations between knowledge, ProQOL, and practice during COVID-19; and 3) to identify the factors associated with nurses’ practice during COVID-19. This study examines how the variables (nurses’ characteristics, knowledge, and ProQOL), in each category, were associated with nurses’ clinical performance, as presented in Fig 1. Thus, the following hypotheses were established (Fig 1):

1. Nurses’ knowledge level has a positive association with practice.
2. Nurses’ characteristics are associated with practice.
3. Nurses’ ProQOL is associated with practice. Specifically, nurses with higher levels of CS and lower burnout and STS engage in better clinical performance.

### Study design/sampling

This was a cross-sectional, descriptive, and correlational study that analyzed the factors associated with nurses’ practice by examining the relationship between their knowledge levels, ProQOL, and practice during the COVID-19 pandemic.

Access to the study participants was obtained after acquiring consent from the nursing director of each medical institution. The inclusion criteria for participating in this study were being registered as a qualified nurse, working in the Republic of Korea, and being willing to participate in the study. Employees outside these parameters, such as nursing students and medical assistants, were excluded. To avoid researcher bias, a questionnaire was distributed to all participants who volunteered and met the inclusion criteria, through the relevant clinical nurse manager, who received a briefing on the study from the appropriate nursing supervisor before distribution of the questionnaire. No incentives were provided, no tips on completing the questionnaire were provided, and participation was anonymous.

### Sample/Participants

The sample size was determined using the G*Power 3.1 program [36]. For a regression analysis with a significance level (α) of .05, power of .95, and eight predictor variables (three general characteristics, knowledge, ProQOL [CS, burnout, STS], and practice), the minimum required sample size was calculated to be 160. Considering a 10% withdrawal rate, data were collected from 175 participants. The inclusion criteria were being over 18 years old, working in patient care at the time of the survey, and completing the questionnaire. After excluding 8 questionnaires with careless and missing responses, 167 were included in the final analysis.

### Data collection

After explaining the purpose and contents of this study to the head of the nursing department and the head of the nursing education team at each hospital, approval was obtained. A researcher was assigned to each hospital and distributed the questionnaire, explained the purpose of the questionnaire, obtained consent to participate, and personally collected the completed questionnaire.

Data were collected from staff nurses, charge nurses, and nurse managers who worked in tertiary, secondary, and national forensic psychiatry hospitals in four cities of the Republic of Korea. Data collection was conducted using a self-report questionnaire during the COVID-19 pandemic, between June and July 2021. The questionnaire that was used to obtain data regarding the participants’ knowledge and practices is presented as S1 Appendix.

### Knowledge and practice

The Knowledge and Practice Toward COVID-19 Scale [37] was used. The knowledge subscale comprises 10 items (causative agent, incubation period, mode of transmission, main symptoms, confirmatory diagnosis, high-risk population for severe outcomes, preventive measures, current management options, possible complications, and mortality rate), each of which is rated from 0 to 10. A score of ≥ 7 indicates adequate knowledge [37].

The practice subscale comprises five items, each of which is rated on a five-point Likert scale. Scores range from 5 to 25, with higher scores indicating better practice. A score of ≥ 18 indicates an appropriate level of practice, and a score of ≤ 17 indicates an inappropriate level of practice [37]. In this study, the Cronbach’s ⍺ for the knowledge and practice subscales were .74 and .72, respectively. Cronbach’s ⍺ was .74 for the overall scale at the time of development.

### ProQOL

Stamm’s Professional Quality of Life Scale Version 5 translated by Kim [38] was used to assess STS, CS, and burnout. The scale comprises CS, burnout, and STS subscales, each of which consists of 10 items that are rated on a five-point Likert scale. The scores for each subscale range from 10 to 50, with higher scores indicating greater CS, burnout, and STS. According to Stamm’s classification, the scores were interpreted as low (≤ 22), moderate (23–41), and high (≥ 42) levels for each corresponding construct. The answers to items 1, 4, 15, 17 and 29 in the STS subscale were reverse coded. The reliability of the subscales (Cronbach’s ⍺) were .88, .75, and .81 for CS, burnout, and STS, respectively, at the time of development, and .93, .75, and .83, respectively, in this study.

### Data analysis

The collected data were analyzed using SPSS 22.0 (IBM Corp., Armonk, NY, USA). The Shapiro–Wilk test was performed to assess the normality of the distribution of continuous variables. Participants’ general characteristics, knowledge, ProQOL, and practice were presented as either frequency and percentage or mean and standard deviation. Differences in practice according to general characteristics were analyzed by performing a t-test and analysis of variance. Correlations between knowledge, ProQOL, and practice were analyzed by calculating Pearson’s correlation coefficients. Further, the associations between practice and general characteristics, knowledge, and ProQOL were analyzed by performing hierarchical regression. Statistical significance was set at p < .05.

### Ethical considerations

This study was conducted in accordance with the Code of Ethics of the World Medical Association (Declaration of Helsinki) for experiments involving humans. After obtaining approval from the institutional review board of Chungnam National University Sejong Hospital (CNUSH 2021-05-017-005), the participants were informed about the purpose and content of the study, anonymity and confidentiality, use of data only for research purposes, voluntary participation, and freedom to withdraw without repercussions at any time. Data were collected from nurses who voluntarily provided written consent.

## Results

### Descriptive statistics

#### Characteristics of the participants

Previous studies [39–43] have shown that infection control education is a significant factor in explaining practice; this study included several variables (e.g. effect of COVID-19 education, understanding of COVID-19) to further refine the factors affecting practice. Most participants (86.2%) were women; the mean age was 31.43 years, with 51.5% aged < 30 years. Further, most participants were single (72.5%) and had a bachelor’s degree (82%). Many participants lived alone (47.9%), and 49.7% had no children. Approximately 70.7% worked at a tertiary hospital, the mean length of employment was 1.90 years, and 48.5% had worked for less than 5 years. The most common monthly income was 2–3 million KRW (65.9%), and the most common work unit was intensive care (33.5%). Moreover, most participants (88.6%) were staff nurses. The vast majority (95.2%) worked rotating shifts, and 56.9% worked 5–6 night shifts per month. Approximately 33.5% were satisfied with their jobs, and 56.3% had provided care for patients with COVID-19. A total of 85.6% had prior COVID-19 management training, and 91.6% were vaccinated against COVID-19. Approximately 85.6% of the participants feared spreading the infection to their families, and 27.6% felt supported by their superiors. Of the participants, 38.3% claimed to be confident in the COVID-19 response, and 53.3% had reduced anxiety about infection after receiving COVID-19 vaccination (Table 1).

**Table 1:** Participants’ characteristics, professional quality of life, knowledge, and practice (N = 167).

Variables	Categories	n (%) or M ± SD	
Gender	Men	23	13.8
	Women	144	86.2
Age (years)	< 30	86	51.5
	30–39	58	34.7
	40–49	18	10.8
	≥ 50	5	3.0
		31.43	± 6.73
Marital status	Unmarried	121	72.5
	Married	46	27.5
Educational level	College	8	4.8
	University	137	82.0
	Graduate school or above	22	13.2
Living arrangement	Alone	80	47.9
	Couple only	15	9.0
	With family	68	40.7
	Roommate	4	2.4
Presence of children	Yes	31	18.6
	No	83	49.7
	Not applicable	53	31.7
Level of hospital	General hospital	29	17.4
	Advanced general hospital	118	70.7
	Institute of Forensic Psychiatry, Ministry of Justice	15	9.0
	Hospital or social welfare facility	5	3.0
Total clinical experience (years)	< 5	81	48.5
	5–10	43	25.7
	10–15	21	12.6
	≥ 15	22	13.2
		1.90	±1.07
Working site	Infection control unit	32	19.2
	Surgical wards	21	12.6
	Emergency room	30	18.0
	Intensive care unit	56	33.5
	Dialysis department	10	6.0
	Medical alert team	4	2.4
	Psychiatry unit	14	8.4
Position	Staff nurse	148	88.6
	Charge nurse or higher	19	11.4
Type of work	Shift work	159	95.2
	Not shift work	8	4.8
Number of night shifts in a month	0	27	16.2
	4	12	7.2
	5–6	95	56.9
	≥ 7	33	19.8
Income (million KRW)	200–300	110	65.9
	300–400	45	26.9
	> 400	12	7.2
Job satisfaction	Strongly disagree	7	4.2
	Disagree	21	12.6
	Moderate	83	49.7
	Agree	53	31.7
	Strongly agree	3	1.8
Experience of working with confirmed cases of COVID-19	Yes	94	56.3
	No	73	43.7
Education on COVID-19 management	Yes	143	85.6
	No	24	14.4
Effect of COVID-19 education	Disagree	5	3.0
	Moderate	30	18.0
	Agree	87	52.1
	Strongly agree	25	15.0
	Not applicable	20	12.0
Understanding of COVID-19	Disagree	10	6.0
	Moderate	79	47.3
	Agree	67	40.1
	Strongly agree	11	6.6
COVID-19 vaccination	Yes	153	91.6
	No	14	8.4
Fear of infection in the family	Yes	143	85.6
	No	24	14.4
Support of supervisor	Strongly disagree	6	3.6
	Disagree	29	17.4
	Moderate	86	51.5
	Agree	40	24.0
	Strongly agree	6	3.6
COVID-19 coping confidence	Disagree	15	9.0
	Moderate	88	52.7
	Agree	54	32.3
	Strongly agree	10	6.0
Reduction of infection anxiety after COVID-19 vaccination	Strongly disagree	3	1.8
	Disagree	21	12.6
	Moderate	43	25.7
	Agree	53	31.7
	Strongly agree	36	21.6
	Not applicable	11	6.6
Experience of dispatch	Yes	65	38.9
	No	102	61.1

n: Number of participants; M: Mean; SD: Standard deviation.

#### Level of knowledge, ProQOL, and practice related to COVID-19

The mean knowledge score was 6.95 ± 1.54 out of 10 (Range: 2–10) (Table 2). Notably, a high percentage of the respondents exhibited good knowledge of COVID-19, recording 94.6% and 92.8% correct answers for the Preventive measures and Incubation period items, respectively. The item with a correct answer rate of less than 50% was Mode of transmission (Table 3). The mean practice score was 21.20 ± 3.69, and 82% demonstrated an appropriate level of practice. The items with the highest and lowest correct answer proportion were preventive measures (94.6%) and causative agent (62.3%), respectively (Table 2).

**Table 2:** Level of knowledge, professional quality of life, and practice related to COVID-19.

	M ± SD	Variables	Categories	n	(%)	M	± SD	Min	Max
Knowledge			Inadequate (< 7)	57	34.1	6.95	1.54	2	10
			Adequate (≥ 7)	110	65.9				
ProQOL	80**±** 11.22 (Min 59 Max 117)	Compassion satisfaction	Low	45	26.9	27.65	7.86	10	50.00
			Moderate	115	68.9				
			High	7	4.2				
		Burnout	Low	18	10.8	29.32	5.94	15	45.00
			Moderate	144	86.2				
			High	5	3.0				
		Secondary traumatic stress	Low	91	54.5	23.03	6.64	10	41.00
			Moderate	76	45.5				
Practice			Inappropriate (≤ 17)	30	18.0	21.20	3.69		
			Appropriate (> 17)	137	82.0				

n: Number of participants; M: Mean; SD: Standard deviation; ProQOL: Professional quality of life.

**Table 3:** Items of knowledge and practice scales related to COVID-19.

Knowledge items	Correct answer	
	**(n)**	**%**
Causative agent	104	62.3
Incubation period	155	92.8
Mode of transmission	66	39.5
Main symptoms	139	83.2
Confirmatory diagnosis	118	70.7
High-risk population for severe outcome	80	47.9
Preventive measures	158	94.6
Current management option	106	63.5
Possible complications	126	75.4
Mortality rate	108	64.7
**Practice items**	**M**	**± SD**
Implementation of five moments of hand hygiene with seven steps	3.95	1.01
Utilization of 60% alcohol-based hand sanitizer in the absence of soap and water	4.39	0.73
Wearing of personal protective equipment	4.26	1.11
Careful doffing of personal protective equipment	4.49	1.00
Isolation of suspected or infected patients	4.11	1.41

n: Number of participants; M: Mean; SD: Standard deviation.

The mean CS score was 27.65 ± 7.86, and 115 nurses (68.9%) had a moderate level of CS. The mean burnout score was 29.32 ± 5.94, and 144 nurses (86.2%) experienced a moderate level of burnout. The mean STS score was 23.03 ± 6.64, and 76 nurses (45.5%) experienced a moderate level of STS (Table 2).

The mean practice score was 21.20 ± 3.69 out of 25 (Table 2). By domain, the score was the lowest for “Implementation of five moments of hand hygiene with seven steps” (3.95 ± 1.01) and the highest for “Careful doffing of personal protective equipment” (4.49 ± 1.00; Table 3).

### Differences in practice according to participants’ characteristics

Infection control unit nurses showed significantly better performance of COVID-19 practices than other ward nurses (F = 13.94, p < .001). The practice scores of nurses with care experience for confirmed cases of COVID-19 (t = 5.67, p < .001), nurses with COVID-19 management education (t = 3.17, p = .004), nurses who perceived the education to be very effective (F = 4.55, p = .002), and nurses who had a very good understanding of COVID-19 (F = 10.04, p < .001) was also found to be significantly high. The practice scores of nurses without fear of COVID-19 infection to the family (t = -2.07, p = .040), nurses who perceived adequate support from their supervisors (F = 2.96, p = .045), nurses with strong confidence for coping with COVID-19 (F = 5.96, p = .003), and nurses with dispatch experience (t = 4.81, p < .001) were significant (Table 4).

**Table 4:** Differences in practice by participants’ characteristics (N = 167).

Variables	Categories	Practice			
		M	± SD	t or F	p
Gender	Men	21.04	4.20	-0.22	.830
	Women	21.22	3.62		
Age (years)	< 30	21.23	3.96	0.78	.509
	30–39	21.43	3.42		
	40–49	21.06	3.21		
	≥ 50	18.80	2.05		
Marital status	Unmarried	21.53	3.63	1.90	.060
	Married	20.33	3.75		
Educational level	College	20.13	3.64	0.35	.703
	University	21.25	3.74		
	Graduate school or above	21.27	3.49		
Living arrangement	Alone	21.43	3.98	0.55	.647
	Couple only	21.87	2.33		
	With family	20.78	3.65		
	Roommate	21.25	2.06		
Presence of children	Yes	20.19	3.84	2.59	.078
	No	21.81	3.38		
	Not applicable	20.83	3.94		
Level of hospital	General hospital	21.86	3.50	2.00	.117
	Advanced general hospital	21.34	3.71		
	Institute of Forensic Psychiatry, Ministry of Justice	19.47	3.44		
	Hospital or social welfare facility	19.20	3.77		
Total clinical experience (years)	< 5	21.37	4.10	0.28	.836
	5–10	21.21	3.31		
	10–15	21.19	3.20		
	≥ 15	20.55	3.38		
Working site	Infection control unit	23.94	1.32	13.94	< .001
	Surgical wards	18.67	4.39		
	Emergency room	22.07	2.78		
	Intensive care unit	20.93	3.96		
	Dialysis department	20.30	3.68		
	Medical alert team	20.50	2.52		
	Psychiatry unit	18.79	2.78		
Position	Staff nurse	21.11	3.82	-0.87	.383
	Charge nurse or higher	21.89	2.45		
Type of work	Not shift work	21.38	2.20	0.14	.890
	Shift work	21.19	3.75		
Number of night shifts in a month	0	21.59	2.71	1.03	.380
	4	19.50	3.06		
	5–6	21.19	3.95		
	≥ 7	21.52	3.78		
Income (million KRW)	200–300	21.45	3.74	2.15	.120
	300–400	20.29	3.70		
	> 400	22.25	2.56		
Job satisfaction	Strongly disagree	19.00	3.87	1.75	.142
	Disagree	20.71	2.70		
	Moderate	21.52	3.78		
	Agree	20.96	3.81		
	Strongly agree	25.00	0.00		
Experience of working with confirmed cases of COVID-19	Yes	22.55	2.79	5.67	< .001
	No	19.45	3.98		
Education on COVID-19 management	Yes	21.64	3.33	3.17	.004
	No	18.54	4.59		
Effect of COVID-19 education	Disagree	18.20	5.17	4.55	.002
	Moderate	21.40	3.68		
	Agree	21.51	3.11		
	Strongly agree	22.52	3.63		
	Not applicable	18.65	4.49		
Understanding of COVID-19	Disagree	20.60	4.70	10.04	< .001
	Moderate	19.99	4.05		
	Agree	22.31	2.71		
	Strongly agree	23.64	1.69		
COVID-19 vaccination	Yes	21.22	3.72	0.28	.777
	No	20.93	3.50		
Fear of infection in the family	Yes	20.96	3.81	-2.07	.040
	No	22.63	2.45		
Supervisor support	Strongly disagree	17.00	5.55	2.96	.045
	Disagree	20.55	3.76		
	Moderate	21.83	3.31		
	Agree	20.65	3.83		
	Strongly agree	23.17	1.94		
Confidence in coping with COVID-19	Disagree	20.67	4.15	5.96	.003
	Moderate	20.32	4.10		
	Agree	22.44	2.41		
	Strongly agree	23.00	2.54		
Experience of dispatch	Yes	22.75	3.08	4.81	< .001
	No	20.21	3.72		
Reduction of infection anxiety after COVID-19 vaccination	Strongly disagree	20.67	6.66	0.33	.894
	Disagree	20.52	4.07		
	Moderate	21.58	3.69		
	Agree	21.40	3.18		
	Strongly agree	21.06	4.21		
	Not applicable	20.64	3.17		

n: Number of participants; M: Mean; SD: Standard deviation.

### Correlations between COVID-19-related knowledge, ProQOL, and practice

Burnout, a domain of ProQOL, was statistically significantly negatively correlated with adherence to guidelines (r = -.184, p = .017), showing that adherence to COVID-19 guidelines increased with decreasing burnout (Table 5).

**Table 5:** Correlations between knowledge, professional quality of life, and practice (N = 167).

	Knowledge	Compassion satisfaction	Burnout	Secondary traumatic stress	Practice
Knowledge	1				
Compassion satisfaction	-0.012 (.877)	1			
Burnout	0.057 (.462)	-.640** (< .001)	1		
Secondary traumatic stress	0.002 (.980)	0.122 (.116)	.404** (< .001)	1	
Practice	-0.001 (.987)	0.143 (.065)	-.184* (.017)	-0.034 (.661)	1

### Predictors of COVID-19-related practice

The Durbin–Watson statistic was 2.003, and the variance inflation factor was 12.259, 10.382, and 9.772 for work units, effects of COVID-19 education, and support from superiors, respectively. Therefore, these three variables were removed from the regression analysis because they showed multicollinearity. Then, the Durbin–Watson statistic was 1.814, and the correlation coefficients were below .67. There was no autocorrelation of the error term, and the tolerance ranged from 0.453 to 0.992, with the variance inflation factor ranging from 1.948 to 5.434, confirming the absence of multicollinearity. Furthermore, there were no sociodemographic factors that statistically significantly varied in relation to practice.

To identify the variables associated with practice, the general characteristics shown to be differently correlated with the practice dimensions were input as independent variables. Model 1 included nurses’ general factors and Model 2 included variables that were significantly different among ProQOL factors; these were input as explanatory variables. The general characteristics that statistically significantly differed in relation to practice—prior COVID-19 care experience, understanding of COVID-19, fear of spreading COVID-19 infection to family, confidence in COVID-19 response, and prior floating experience—were entered as independent variables. Model 1’s regression equation was statistically significant (F = 6.27, p < .001) and explained 24.1% of the variance in COVID-19 practice. Prior COVID-19 education was identified as a predictor (t = 2.49, p = .014). In Model 2, burnout, which differed statistically significantly according to practice, was also entered. Model 2’s regression equation was also statistically significant (F = 6.79, p < .001), and the percentage of explained variance increased by 3.8% to 27.7%. Burnout was identified as a statistically significant predictor of clinical practice (Table 6).

**Table 6:** Multiple regression results for practice (N = 167).

Variables	Model 1				Model 2			
	B	SE	Β	p	B	SE	β	p
Constant	19.765	1.420		< .001	24.120	2.018		< .001
Fear of infection in the family (Yes)	-0.645	0.736	-.062	.382	-0.470	0.720	-.045	.515
Experience of working with confirmed cases of COVID-19 (Yes)	1.270	0.745	.171	.090	1.832	0.751	.247	.016
Education on COVID-19 management	1.936	0.778	.185	.014	1.635	0.766	.156	.034
Understanding of COVID-19 (Moderate)	-1.225	1.161	-.166	.293	-1.306	1.133	-.177	.251
Understanding of COVID-19 (Agree)	0.385	1.246	.051	.758	0.327	1.216	.044	.788
Understanding of COVID-19 (Strongly agree)	1.231	1.712	.083	.473	1.003	1.672	.068	.549
COVID-19 coping confidence (Moderate)	-0.808	0.956	-.110	.400	-1.317	0.949	-.179	.167
COVID-19 coping confidence (Agree)	-0.036	1.092	-.005	.973	-0.733	1.091	-.093	.503
COVID-19 coping confidence (Strongly agree)	-0.383	1.603	-.025	.811	-1.239	1.591	-.080	.437
Experience of dispatch (Yes)	1.070	0.712	.142	.135	0.852	0.698	.113	.224
Burnout					-0.132	0.044	-.212	.003
Adj R^2^	0.241				0.277			
ΔR^2^					0.038			
F (p)	6.273	< .001			6.790	< .001		

SE: Standard error.

## Discussion

In this study, 65.9% of the participants displayed an adequate level of knowledge. This is similar to previous findings that non-physician healthcare professionals, including nurses, demonstrated inadequate knowledge of optimal infection prevention measures against COVID-19 [35,38] and infection routes during the Middle East Respiratory Syndrome (MERS) epidemic [37,43]. Further, among care behaviors, “knowledge and skills” received the lowest score [44], contradicting a report that this group demonstrated a high level of COVID-19-related knowledge, including with regard to disease symptoms [32,44]. The diversity of study populations, varying content of scales measuring knowledge by domain, and different evaluation criteria probably influenced the results. However, given that the study population primarily comprised nurses working in hospitals designated to manage patients with COVID-19, who lacked knowledge about the route of COVID-19 transmission, continued COVID-19 education with up-to-date information and evidence-based knowledge that addresses the characteristics of the disease is essential.

Similar to our study, another study [45] showed that healthcare providers did not have sufficient knowledge of the signs, transmission, prevention, and treatment of COVID-19. In El‐Monshed et al.’s study [46] the proportion of correct answers was higher than 85% during the main symptom and incubation period, similar to the results of this study.

The current participants showed a moderate level of CS, burnout, and STS. Specifically, 73.1% demonstrated a moderate or high level of CS, a positive construct, with the percentage of participants with moderate or low levels of burnout and STS, which are negative constructs, at 97% and 100%, respectively. Similar results were reported by a study of emergency department nurses before the COVID-19 pandemic [47], a study of US nurses [48], a study of intensive care unit and isolation ward nurses after the outbreak of COVID-19 [49], and a study of Wuhan nurses [50]. This is different from a study with Chinese nurses, in which CS was moderate, and burnout and STS were low [51]. The study results may differ depending on the situation in each country, hospital, or work environment. It has been reported that providing a supportive work environment [52], reasonable shift schedules for limiting nurses’ work hours [53], reducing burnout among nurses, and providing psychological counseling services may affect STS [50]. The findings highlight the need for systems or policies that boost CS and reduce STS and burnout to enhance the efficiency of nursing work and quality of care, regardless of COVID-19.

Approximately 82% of the participants displayed appropriate practice, similar to previous findings that 78.9% of healthcare workers demonstrated appropriate practice [35] and that nurses “frequently” or “always” adhered to the isolation guidelines (score 3.34/4) during the MERS epidemic [29,43]. Although handwashing is an effective means to prevent the spread of infection [45,54], the practice score for this domain was the lowest (3.95) in our study, thus calling for continued education and monitoring.

Knowledge and practice were not significantly correlated in this study, similar to previous findings [46,47,55,56]. However, this contrasts with the findings of Zhang et al. [11]. Although this study was conducted during the delta variant wave, 34.1% of the participants demonstrated an inappropriate level of knowledge, and the mean knowledge score was also below the cutoff set by the instrument developer. Increasing knowledge and awareness of infection can facilitate better practice, such as the correct use of personal protective equipment, which can help halt the spread of infection [48,57]. In this study, it was observed that prior COVID-19 response education and training positively contributed to practice despite a low knowledge score. Therefore, COVID-19 education should include basic content, such as mode of transmission and high-risk populations for severe outcomes.

Job-related factors that significantly influenced practice were working in the infectious disease ward, prior COVID-19 care, prior education, effects of education, understanding of COVID-19, fear of spreading COVID-19 to family, support from superiors, confidence in COVID-19 response, and prior floating experience. These results are similar to those of previous studies that identified working in an infectious disease ward, prior COVID-19 or MERS care experience, prior education, and understanding of education as predictors of nurses’ adherence to isolation guidelines during the MERS epidemic [37] and COVID-19 pandemic [47,56]. It seems that nurses working in infection isolation wards follow the guidelines more strictly to protect themselves and provide better patient care [49,58]. COVID-19 education and prior COVID-19 care experience also seem to boost nurses’ understanding of the disease and help follow isolation guidelines more proficiently [50,59].

Regardless of exposure status, healthcare professionals who provided care for patients admitted via an ambulance or older adults with multiple comorbidities during the COVID-19 pandemic showed greater fear of infecting their families [51,52,60]. Further, during the 2009 H1N1 pandemic, fear that their families would die from the virus was associated with lower willingness to work [53,61]. Therefore, the level of practice in our study population may have been influenced by the participants’ high level of fear of spreading the infection to their families, as the sample comprised many nurses working in units that care for vulnerable populations who can readily experience negative outcomes (complications) from COVID-19 infection, such as patients in the emergency department, intensive care unit, and infectious disease wards.

Similar to our findings, previous studies reported that support from superiors [54,62] and confidence in response measures [11] significantly influenced nursing practice during an infectious disease outbreak. Support from superiors, such as sharing of information, provision of education, and monitoring of nursing practice, may motivate nurses to adhere to safety protocols and commit to their jobs and boost their confidence in their response capability during the pandemic, positively influencing their practice.

However, studies examining the association between floating and practice are lacking. During the COVID-19 pandemic, nurses who were transferred to a different unit had a high fear of infection and showed a significantly greater prevalence of depression and anxiety [55,63]. Such negative emotions may adversely impact their work stress, job satisfaction, patients and their families, and organization by decreasing their work efficiency [56,64]. Conversely, a previous study [57,65] reported that depression and anxiety are not significantly associated with nurses’ practices, such as handwashing and use of personal protective equipment, calling for further research on the relationship between floating and practice.

Hierarchical regression was performed to identify the predictors of nurses’ practice. Model 1, which comprised job-related factors, explained 24.1% of the variance in practice, and prior COVID-19 education was the key predictor. Ramirez-Baena et al. [66] reported that training and education about the etiology of a novel infectious disease, infection prevention, and infection control might enhance nurses’ knowledge and skills and that prior COVID-19 education seems to have had a positive influence on nurses’ practice in this study. These results are contrary to those of a study on healthcare professionals [67] However, this could be attributed to the difference in the proportion of participants with prior education in the said (68.9%) and current study (85.6%).

Model 2 explained 27.7% of the variance, and burnout was identified as another significant predictor of practice, in addition to COVID-19 education. Previous studies have reported that low burnout is associated with better hand hygiene practices [68]. Healthcare professionals providing HIV care are more likely to engage in unprofessional behaviors and provide suboptimal care (shouting at patients, not performing diagnostic tests owing to a desire to finish quickly) when they are burned out [69]. Further research is needed on burnout and practice to gain a more accurate understanding of the effects of burnout on practice during an epidemic of an unknown infectious disease, such as COVID-19.

### Limitations

This study has some limitations. First, the study population, which included selected hospitals, was not representative of the entire nursing population, which diminishes the generalizability of the findings. Second, this was an observational, cross-sectional study. Therefore, causality could not be firmly established, and changes over time could not be observed. Third, the identified predictors explained < 30% of the total variance in each model. Additional studies are needed to measure other nursing work environment factors (appropriateness of staffing and workload, support from superiors, and patient acuity) and analyze their associations with practice.

Despite these limitations, this study comprehensively examined nurses’ knowledge, ProQOL, and practice. Unlike previous studies that only addressed nurses’ knowledge, attitudes, and practice, the findings of this study could help establish strategies to prevent the spread of infectious diseases.

## Conclusions

In this study, the aim was to examine the knowledge of COVID-19 isolation guidelines, ProQOL, and practice among nurses and identify the predictors of practice. The participants demonstrated an inadequate level of COVID-19-related knowledge, moderate ProQOL, and an appropriate level of practice. Prior COVID-19 education and burnout were significant predictors of practice. These results highlight the need to establish an effective prevention system for infectious diseases such as COVID-19 by implementing policies and other measures, including education programs pertinent to the prevention and management of infection that improve the modifiable predictors of nurses’ practice—education and burnout.

### Implications for practice

This study revealed an unprecedented result, in that while clinical nurses’ level of knowledge about COVID-19 did not affect their practice during the pandemic, which was a time of continued uncertainty, a high level of knowledge was positively associated with practice. Therefore, educational programs focusing on knowledge that can enhance the practical aspects of nursing are needed, rather than theoretical knowledge.

Exhaustion was negatively associated with practice. In an infectious disease epidemic, nurses’ practice can have a negative impact on patient safety and care quality. Therefore, support systems, including psychological support, which can reduce nurses’ burnout, are essential for nursing managers and nursing leaders, and should be considered in governmental policies.

Although the study collected data after vaccination against COVID-19 and relaxation of precautions, most participants experienced a moderate level of burnout, and 45.5% of participants experienced a moderate level of STS. Burnout was a significant factor affecting nurses’ performance. Regardless of the COVID-19 pandemic, counseling and education programs and feedback that can reduce nurse burnout and STS and improve practical performance are essential from a policy point of view.

Based on the present results, education on COVID-19 management is positively associated with practice, suggesting that education is an important strategy by which to improve practice. Considering that the proportion of correct answers was low for basic knowledge, such as causes of COVID-19, mortality, and management, systematic education including basic knowledge and testing of the effectiveness of education are also necessary.

Leaders and managers of healthcare institutions must strive to identify the causes of burnout among nurses, and professionals must develop strategies to reduce burnout by increasing CS and reducing STS, so that practice can be improved.

## Supporting information

S1 Checklist STROBE statement—checklist of items that should be included in reports of observational studies. (DOCX)

S1 Appendix Questionnaire for assessing knowledge and practices. (DOCX)
