# Supplementary material for: Relationship between nurses’ knowledge of COVID-19, professional quality of life, and practice during the COVID-19 pandemic: A descriptive correlational study
Source: PLoS One. 2023 Jun 22;18(6):e0287457. doi: 10.1371/journal.pone.0287457 (PMC10286984; doi:10.1371/journal.pone.0287457)
Supplement: S1 Appendix — (DOCX) [file pone.0287457.s002.docx]

**S1 Appendix. Questionnaire for assessing knowledge and practices**

**Knowledge (**single response possible**):-**

1. The virus causing COVID-19 is…
   - SARS-CoV
   - MERS-CoV
   - SARS-CoV-2
   - Ebola Virus

The incubation period of COVID-19 is…

- - 2 to 14 days
  - 2 to 11 days
  - 2 to 21 days
  - 7 to 21 days

1. The mode of transmission of COVID-19 is…
   - Respiratory droplets
   - Direct (infected person) and indirect contact (surface, objects such as thermometers)
   - Airborne transmission (aerosol)
   - All of the above
2. The main symptom of COVID-19 is …
   - High-grade fever
   - Dry cough
   - Shortness of breath
   - All of the above
3. The confirmatory diagnosis for COVID-19 is …
   - Rapid diagnostic test
   - Reverse transcription – polymerase chain reaction
   - Chest computed tomography scan
   - All of the above
4. The high-risk population for severe COVID-19 outcomes is …
   - Children
   - Pregnant women
   - Older adults (over 65 years) and people with underlying medical conditions such as diabetes, heart disease, kidney disease, asthma, and cancer
   - All of the above

Preventive measures for COVID-19 infection include ...

- - Handwashing with soap and water
  - Social distancing and isolation of suspected and confirmed cases
  - Wearing personal protective equipment while caring for infected patients
  - All of the above

1. The management option for COVID-19 is …
   - Supportive and symptomatic management
   - Rehabilitative management
   - Palliative care
   - Antiviral therapy
2. Complications of COVID-19 include …
   - ARDS
   - Shock
   - Organ dysfunction (acute kidney injury, acute lung injury, acute liver injury, acute cardiac injury, etc.)
   - All of the above
3. The mortality rate of COVID-19 is…
   - 0.5 to 3%
   - 9.5%
   - 34.4%
   - 39%

**Practice (**single response possible**): -**

1. I follow five moments of hand hygiene with the seven steps.
   - Always
   - Often
   - Sometimes
   - Rarely
   - Never
2. I use 60% alcohol-based hand sanitizer in the absence of soap and water.
   - Always
   - Often
   - Sometimes
   - Rarely
   - Never
3. I wear personal protective equipment while caring for patients.
   - Always
   - Often
   - Sometimes
   - Rarely
   - Never
4. I carefully remove personal protective equipment and discard it in the proper place.
   - Always
   - Often
   - Sometimes
   - Rarely
   - Never
5. We isolate suspected and/or infected patients.
   - Always
   - Often
   - Sometimes
   - Rarely
   - Never
